# Pubertal development is unaffected in female *Mecp2* -heterozygous mice.

**DOI:** 10.17912/micropub.biology.002400

**Published:** 2026-09-09

**Authors:** Skylar Fortich, Lily Demilio, Mariam Ceesay, Anabelle Clower, Billy You Bun Lau

**Affiliations:** 1 University of Tennessee, Knoxville, TN, United States; 2 Psychology & Neuroscience, University of Tennessee, Knoxville, TN, United States

## Abstract

Rett syndrome (RTT) affects predominantly females and is characterized by regression after an initial period of typical development. Previously, we showed that while adolescent female *Mecp2* -heterozygous mice (Het, mouse model of RTT) perform pup retrieval comparably to wild-type littermate controls (WT), adult Het regress and become inefficient. Here, we analyzed the pubertal trajectory of naïve nulliparous WT and Het to test whether abnormal pubertal development contributes to this regression phenotype. We found no genotypic differences in pubertal onset nor estrous cyclicity during adolescence and adulthood. These findings further support the role of *Mecp2* in experience-dependent plasticity within sensory cortices.

**Figure 1. Pubertal onset and estrous cycling are not affected by Mecp2 mutations:** **A.** Schema of experimental design. Upon vaginal opening, animals were lavaged daily up to postnatal day 55 (P55), and lavaged again from P84-98. Cytology and imaging were performed to determine estrous phase. **B.** Example bright field images showing proestrus, estrus, metestrus and diestrus. Black arrows = nucleated epithelial cells, black arrowheads = cornified epithelial cells, white arrows = leukocytes. **C-D.** Vaginal opening (puberty onset; C) and first estrus (D) were not significantly different between WT (n=28, 9, respectively) and Het (n=35, 9, respectively). **E-H.** There were no significant differences between WT and Het in average cycle length during adolescence (E) and adulthood (G) nor in % time spent in the 4 different estrous phases during adolescence (F) and adulthood (H). E-F: WT (n=8) and Het (n=8). G-H: WT (n=10) and Het (n=9). C-H: Scatter plots: each dot represents an animal, lines are mean ± S.E.M. *Mann-Whitney test: p>0.05,* n.s. = no significance.

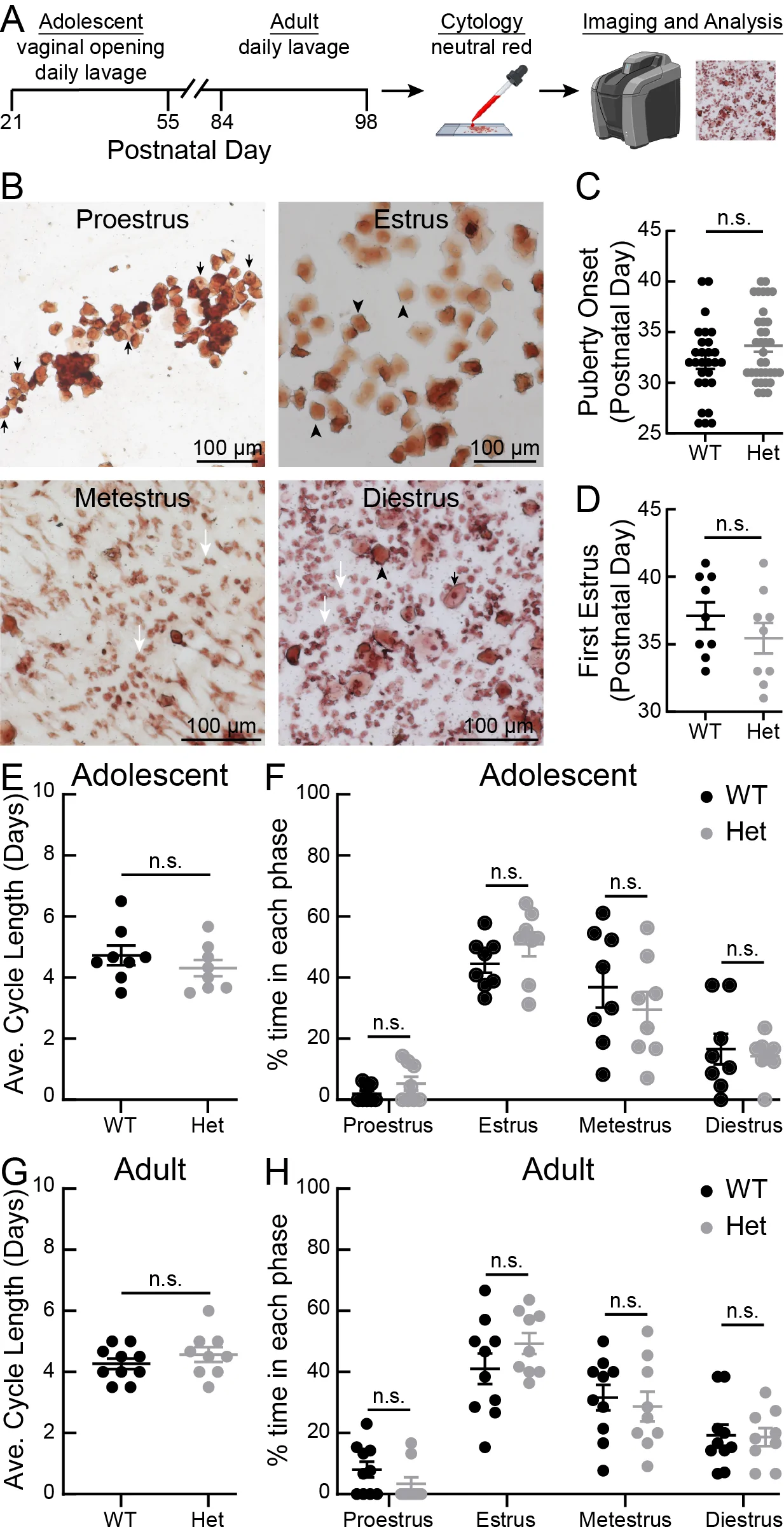



## Description

RTT is a rare neurodevelopmental disorder that predominantly affects girls, with a prevalence of 1 in 10,000 live female births. It is caused by mutations in the X-linked gene, methyl-CpG-binding protein 2 ( *MECP2* ) (Amir et al., 1999). RTT patients exhibit typical development until 6-18 months of age, when they experience developmental stagnation followed by a period of regression. Regression is characterized by the loss of previously acquired skills, such as purposeful hand movement, communication skills and motor function, that persists throughout life (Neul et al., 2010; Halbach et al., 2013). Pubertal abnormalities have also been reported in RTT patients. For instance, Killian et al. (2014) sampled 802 RTT patients and reported that 25% exhibited premature thelarche, 28% premature adrenarche and 19% delayed menarche. A subset of RTT patients with milder mutations had less severe phenotype and earlier menarche. Another example was presented in a case study by Holm (1985), where the RTT patient exhibited precocious puberty at the age of 6, and subsequent case studies documented similar findings (Bas et al., 2013; Yang et al., 2021; Canton et al., 2023). Pubertal dysfunction in RTT is further underscored by the high prevalence of catamenial seizures (Humphrey et al., 2021). Despite substantial evidence of linking RTT with abnormal pubertal and reproductive phenotypes, whether these abnormalities contribute to regression remains unclear.

Previously, we identified a regression phenotype in a mouse model of RTT, female *Mecp2* -heterozygous mice (Het; Guy et al., 2001). At 6 weeks old (adolescent), Het perform as efficiently as wild-type littermate controls (WT) in an alloparental pup retrieval behavior (Mykins et al., 2023). By 12 weeks old, (adult), pre-symptomatic Het regress and perform the same task less efficiently than WT (Krishnan et al., 2017; Stevenson et al., 2021; Mykins et al., 2024). One possible explanation for the Het regression phenotype is the abnormal pubertal development caused by *Mecp2* mutations. Here, we tested this hypothesis by examining pubertal onset and estrous cyclicity in adolescent and adult mice. Pubertal onset was determined by the day of vaginal opening, and estrous cyclicity was determined by daily vaginal lavage followed by cytological analysis ( **Figure 1A** ). The mouse estrous cycle is divided into 4 phases distinguished by their distinct cellular composition (McLean et al., 2012): proestrus = mostly nucleated epithelial cells, with some cornified epithelial cells; estrus = mainly cornified epithelial cells; metestrus = mostly leukocytes, stringy tissue may be present; and diestrus = mostly leukocytes, with some cornified and nucleated epithelial cells ( **Figure 1B** ). We found that puberty onset was similar between WT and Het ( **Figure 1C ;** WT: 32.1 ± 0.7 days, n = 28 animals; Het: 33.7 ± 0.6 days, n = 35 animals; *Mann-Whitney: p>0.05* ). In a subset of those mice, we determined the day of first observed estrus, an indirect indicator of reproductive maturation, and we also found no difference between WT and Het ( **Figure 1D ;** WT: 37.1 ± 1.0 days, n = 9 animals; Het: 35.44 ± 1.1 days, n = 9 animals; *Mann-Whitney: p>0.05* ). To determine the phases of estrous cycle, cytological images were scored by 3 experimenters who were blinded to the genotypes. The human inter-rater agreement was 88.98%, consistent with field standards (Caligioni, 2009). In adolescence, there were no significant differences between the genotypes for their average estrous cycle length ( **Figure 1E** ) (WT: 4.7 ± 0.3 days, n = 8 animals; Het: 4.3 ± 0.3 days, n = 8 animals; *Mann-Whitney test: p=0.42* ) nor their percentage of time spent at the 4 different phases ( **Figure 1F** ) (WT: proestrus - 2.0% ± 1.0, estrus - 44.5% ± 2.9, metestrus - 36.9% ± 6.7, diestrus - 16.6% ± 5.0, n = 8 animals; Het: proestrus - 5.3% ± 2.2, estrus – 50.9% ± 4.0, metestrus – 29.5% ± 5.8, diestrus: 14.3% ± 2.4 , n = 8 animals; *Mann-Whitney test: p>0.05* ). In adults, we also did not find significant differences between WT and Het in their average estrous cycle length ( **Figure 1G** ) (WT: 4.2 ± 0.2 days, n = 10 animals; Het: 4.6 ± 0.2 days, n = 9 animals; *Mann-Whitney test: p=0.41* ) nor percentage of time spent at the different phases ( **Figure 1H** ) (WT: proestrus - 8.0% ± 2.6, estrus – 41.0% ± 5.0, metestrus – 31.6% ± 4.2, diestrus – 19.3% ± 3.5, n = 10 animals; Het: proestrus – 3.3% ± 2.2, estrus – 49.3% ± 3.5, metestrus – 28.7% ± 4.9, diestrus: 18.7% ± 3.0, n = 9 animals; *Mann-Whitney test: p>0.05* ).

Taken together, our results indicate that our female mouse model of RTT, female *Mecp2* -heterozygous mice (B6.129P2(C)- *Mecp2 ^tm1.1Bird/J^* ), exhibits normal pubertal development and maturation of the endocrine system during the pre-symptomatic age. Our findings are consistent with those reported in another female mouse model of RTT (Martin-Sanchez et al., 2026). The same study showed that symptomatic *Mecp2* -knock out male mice exhibit delayed pubertal onset, supporting the hypothesis that neuroendocrine alterations may emerge as female *Mecp2* -heterozygous mice age and become symptomatic. Our data further support the idea that the Het regression phenotype in pup retrieval behavior is unlikely to result from gross systemic endocrine disruption and is instead more consistent with age-dependent abnormalities in cellular and circuit plasticity within sensory cortices (Krishnan et al., 2017; Lau et al., 2020a; Lau et al., 2020b).    Nevertheless, the absence of overt abnormalities in pubertal timing or estrous cyclicity does not exclude the possibility that more subtle neuroendocrine mechanisms contribute to age-dependent regression in Het. Future studies will be needed to determine whether *Mecp2* mutations alter hormone-dependent modulation of maternal behavior and experience-dependent plasticity at the level of specific neural circuits.

## Methods

*Animals*

All experiments were performed in female mice (4-16 weeks old), maintained on a 12/12 h light/dark cycle (lights on 7 A.M.) and received food ad libitum. Genotypes used were *Mecp2* -heterozygote (Het; B6.129P2(C)- *Mecp2 ^tm1.1Bird/J^* , JAX # 003890) and *Mecp2* -WT siblings. All procedures were conducted in accordance with the National Institutes of Health’s Guide for the Care and Use of Laboratory Animals and approved by the University of Tennessee-Knoxville Institutional Animal Care and Use Committee.

*Pubertal onset and estrous cyclicity*

Pubertal onset was evaluated by daily visual inspection for vaginal opening (VO), starting at postnatal day 21 (P21). Beginning on the day of VO, mice were lavaged daily for at least 2 weeks (adolescent age) to determine estrous cycle length and their relative duration in each cycle phase. The first detected estrus was used as the cycle-start marker. To assess estrous cyclicity in adulthood, 12-week-old mice (P84) underwent daily vaginal lavage for 2 weeks.

*Cytology, Imaging and Phase Classification*

Vaginal cells were collected by gently flushing the vaginal canal with 50–100 µL Milli-Q water using a glass eyedropper. The fluid was expelled and recollected in a single lavage, and this procedure was not repeated more than twice per session to avoid inducing pseudopregnancy. Samples were placed onto slides and air-dried on a hot plate (~50°C) for 2-3 minutes. Slides were then stained for 2-3 minutes with 0.1% Neutral Red (Sigma-Aldrich, #72210) dissolved in Milli-Q water, gently rinsed with distilled water and air-dried before imaging. Samples were imaged as brightfield montages using a Keyence microscope at 20x magnification. Cyclic phases - proestrus, estrus, metestrus and diestrus - were visually determined from the images by at least three human observers blinded to genotype. Phases were identified based on their characteristic cellular compositions, including nucleated epithelial cells, cornified epithelial cells and leukocytes. The human inter-rater agreement averaged 88.98% across 433 total phase counts, which is consistent with field standards (Caligioni, 2009).

*Statistical Analysis*

All statistical analyses and graphs were performed in GraphPad Prism 11. Mann-Whitney test was used to compare WT and Het. Statistical significance was defined at p < 0.05. Data are presented as mean ± standard error mean (S.E.M.). The figure was generated in Adobe Illustrator 2025.
