# Erratum: Pubertal development is unaffected in female *Mecp2* -heterozygous mice.

**DOI:** 10.17912/micropub.biology.002442

**Published:** 2026-09-28

## Description

The journal missed an author request to correct their funding statement before indexing of this article. 

The funding statement has now been updated from 

"The author(s) declared that financial support was received for this work and/or its publication. This work was supported by UTK startup fund awarded to BYBL, UTK Faculty Research Assistants Funding awarded to BYBL to support SF and LD, and UTK Advanced Undergraduate Research Activity awarded to SF, LD and AC. Funding for open access to this research was provided by University of Tennessee's Open Publishing Support Fund." 

to

"The author(s) declared that financial support was received for this work and/or its publication. This work was supported by UTK startup fund awarded to BYBL, UTK Faculty Research Assistants Funding awarded to BYBL to support SF and LD, and UTK Advanced Undergraduate Research Activity awarded to SF, LD and AC. Funding for open access to this research was provided by University of Tennessee's Open Publishing Support Fund. Funding for open access to this research was provided by University of Tennessee's Open Publishing Support Fund."

